# Call to action: overcoming enrollment disparities in cancer clinical trials with modernized eligibility criteria

**DOI:** 10.1093/jncics/pkad009

**Published:** 2023-02-21

**Authors:** Andrea N Riner, Devon C Freudenberger, Kelly M Herremans, Vignesh Vudatha, Daniel W Neal, Thomas J George, Jose G Trevino

**Affiliations:** Department of Surgery, University of Florida College of Medicine, Gainesville, FL, USA; Department of Surgery, Virginia Commonwealth University School of Medicine, Richmond, VA, USA; Department of Surgery, University of Florida College of Medicine, Gainesville, FL, USA; Department of Surgery, Virginia Commonwealth University School of Medicine, Richmond, VA, USA; Department of Surgery, University of Florida College of Medicine, Gainesville, FL, USA; Department of Medicine, Division of Hematology and Oncology, University of Florida College of Medicine, Gainesville, FL, USA; Department of Surgery, Virginia Commonwealth University School of Medicine, Richmond, VA, USA; Massey Cancer Center, Virginia Commonwealth University School of Medicine, Richmond, VA, USA

## Abstract

Traditional clinical trial eligibility criteria restrict study populations, perpetuating enrollment disparities. We aimed to assess implementation of modernized eligibility criteria guidelines among pancreatic cancer (PC) clinical trials. Interventional PC trials in the United States since January 1, 2014, were identified via clinicaltrials.gov with December 31, 2017, as the transition for pre- and postguidance eras. Trials were assessed for guideline compliance and compared using Fisher exact test. In total, 198 trials were identified: 86 (43.4%) were pre- and 112 (56.6%) postguidance era. Improvements were seen in allowing patients with history of HIV (8.6% vs 43.8%; *P* < .0001), prior cancer (57.0% vs 72.3%; *P* = .034), or concurrent and/or stable cancer (2.1% vs 31.1%; *P* < .0001) to participate. Most (>95%) trials were compliant with laboratory reference ranges, QT interval corrected for heart rate (QTc) cutoffs, and rationalizing excluding prior therapies both pre- and postguidance eras. However, overall compliance with modernized criteria remains poor. We advocate for stakeholders to update protocols and scrutinize traditionally restrictive eligibility criteria.

Clinical trial eligibility criteria define the study population and reduce patient risk. However, traditional criteria restrict populations to the healthiest patients, perpetuating disparities in enrollment and disproportionately excluding Black patients (1). In 2017, the American Society of Clinical Oncology, Friends of Cancer Research, and the US Food and Drug Administration issued guidance on modernized study criteria to influence generalizability of results. Guidelines liberalized criteria for performance status, HIV, organ dysfunction, prior and current malignancies, comorbidities, prior therapies, washout periods, concomitant medication, and brain metastases (2-8). The extent to which these criteria have been implemented remains unknown, and importantly, their impact on clinical trial participation must be investigated (9).

We investigated the use of modernized eligibility criteria in clinical trials pre- and postpublication of updated guidance. We selected pancreatic cancer (PC), where outcomes are poor, trials are ample, and enrollment fulfills unmet clinical needs, and we previously showed that modifying criteria achieved more equitable enrollment (1). Interventional PC clinical trials in the United States starting January 1, 2014, or later in clinicaltrials.gov were identified. Trials were excluded if they were international, as the guidelines are US-based, addressed other malignancies, or were noninterventional. Trials were assessed for compliance with December 31, 2017, as the transition for pre- and postguidance eras to include 4 years of data in both. Only clinicaltrials.gov was referenced for 13 specific criteria, as this is a common source of information for referring physicians and patients. Fisher exact test was used to compare trials by disease stage, year, study phase, and sponsor to determine their influence in following modernized guidelines. Analyses were 2-sided using α = 0.05 and conducted using R statistical software (version 3.6.3; R Foundation for Statistical Computing, Vienna).

In total, 198 trials were identified: 86 (43.4%) were pre- and 112 (56.6%) postguidance era (Figure 1). In the postguidance era, improvements were seen in allowing HIV+ patients (43.8% vs 8.6%; *P* < .0001) and patients with prior cancer (72.3% vs 57.0%; *P* = .034) or concurrent, yet stable, cancer (31.1% vs 2.1%; *P* < .0001) to participate in trials (Table 1). Improved compliance was a function of time, unrelated to disease stage, study phase, or sponsor (Supplementary Table 1, available online). Nearly all (>95%) trials were compliant with using reference ranges for laboratory tests, QTc cutoffs, and rationale for excluding prior therapies in both eras. Modernized criteria recommendations for cardiac function measurements, cardiac abnormalities, time-based washout periods, full recovery from prior nonclinically relevant adverse events, and stable brain metastases were not statistically significantly altered.

**Figure 1.**
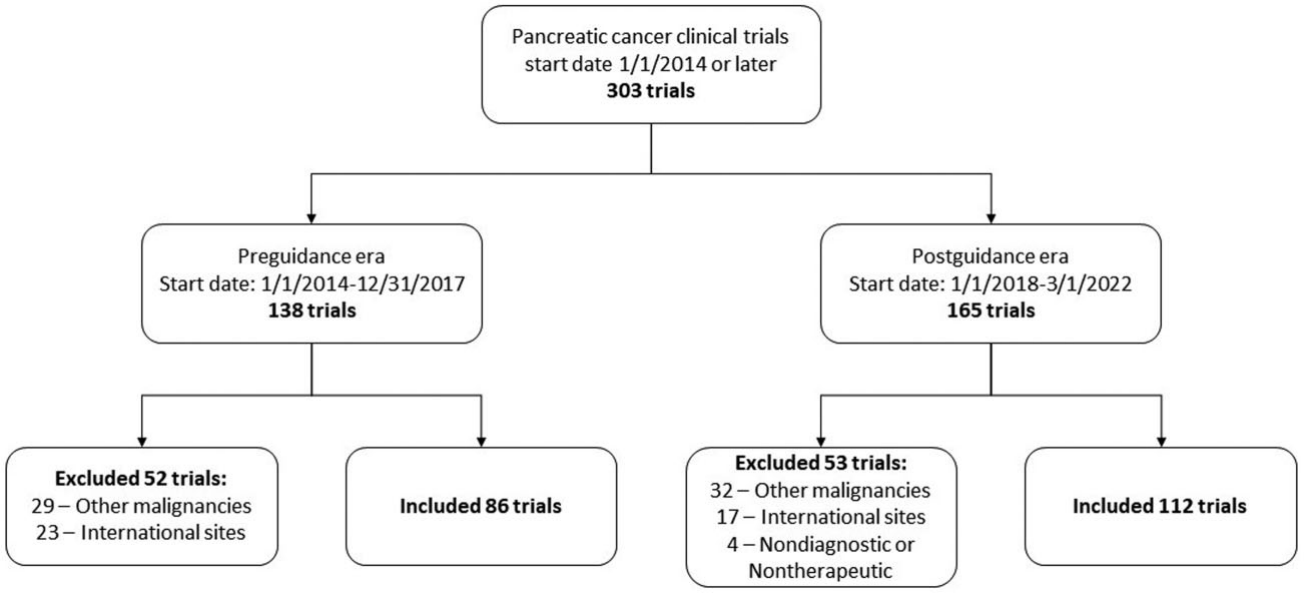
Inclusion and exclusion of interventional pancreatic adenocarcinoma trials conducted in the United States.

**Table 1.** Compliance with modernized eligibility criteria pre- and postrelease of American Society of Clinical Oncology, Friends of Cancer Research, and US Food and Drug Administration guidelines in late 2017^a^

Eligibility criteria	Pre (n = 86, 43.4%) n/N (%)	Post (n = 112, 56.6%) n/N (%)	*P*
Performance status of 2 allowed	27/74 (36.5)	38/105 (36.2)	1.000
HIV+ allowed	5/58 (8.6)	49/112 (43.8)	<.0001
Renal function compliant (use of creatinine clearance (CrCl) only)	6/62 (9.7)	7/73 (9.6)	1.000
Reference ranges used for labs assessing organ function	57/59 (96.6)	72/75 (96.0)	1.000
Classification system used for assessing heart failure	26/49 (53.1)	35/61 (57.4)	.702
Cardiac abnormalities specified	59/86 (68.6)	67/112 (59.8)	.234
QTc compliant	70/74 (94.6)	106/109 (97.2)	.443
Prior malignancy >24 mo ago allowed	49/86 (57.0)	81/112 (72.3)	.034
Concurrent malignancy allowed if stable and off treatment	1/47 (2.1)	23/74 (31.1)	<.0001
Prior therapy allowed + exclusions must be specified	56/56 (100)	71/75 (94.7)	.135
Time-based washout period used	33/55 (60.0)	41/77 (53.2)	.480
Mentions recovery from prior adverse events	25/55 (45.5)	29/77 (37.7)	.377
Brain metastases allowed if stable >4 wk	21/41 (51.2)	25/37 (67.6)	.171

a Denominators vary based on availability of data; missing or unknown values were not included in determining the proportion of trials that were compliant. Allowance of brain metastases, if stable for >4 weeks, was assessed only for trials that included patients with metastatic disease.

With minimal improvement, the current implementation of modernized eligibility criteria allows for increased participation of only a select few patients while continuing to fall short of increasing inclusivity. For example, HIV status contributes to disparities in eligibility of Black patients (1). Although including HIV patients may improve eligibility for Black patients, its low prevalence compared with other comorbidities is striking. In the United States, approximately 1.2 million people have HIV and 407 100 are aged 55 years or older, of which 164 010 are Black individuals (10). Taken in context with PC incidence (11), including HIV+ patients potentially affects 54 patients, 26 of whom are Black individuals. Thus, PC clinical trial inclusivity is impacted minimally through liberalizing HIV criteria.

There is opportunity for improvement with other comorbidities. Chronic kidney disease afflicts 37 million people, and Black individuals are 3 times more likely to be impacted (12). It may be as simple as removing creatinine, a controversial kidney function measure, and instead using glomerular filtration rate (13). However, only 10% of trials followed renal function recommendations, which is unacceptably low given the long-standing guidance that glomerular filtration rate estimates should be used because of creatinine’s disparate impact on minority populations.

Improved compliance with modernized criteria was associated with time but not disease stage, study phase, or sponsor. Patients with advanced disease seeking palliation were subjected to the same criteria as patients with curable disease. Phase III studies are not associated with more modernized criteria relative to early phase studies where safety determinations could arguably be more critical. Finally, the lack of association with trial sponsor is reflective of an issue across clinical research. Lack of compliance with modernization is pervasive, indicating the need for revolutionizing how patients are assessed for participation.

This begs the question of why we continue to fall short. It’s possible that protocols and historical criteria carried forward, lacking knowledge or consideration of modernized criteria. However, there may be other reasons for slow uptake including concerns that agencies may not find changes acceptable or precedent hasn’t been established. Such decisional paralysis supports the need for stakeholder accountability and criteria change in trials where appropriate.

This study was limited by referencing criteria from clinicaltrials.gov and not directly from study protocols, potentially limiting the accuracy of listed criteria. Discrepancies can exist between criteria in a study’s protocol and listing, which may lead to misrepresentation of criteria compliance (14). Furthermore, trials were considered HIV compliant if explicitly stated or lacked exclusion of serious infections requiring systemic therapy, possibly overestimating true compliance. Additionally, uptake of guidelines is likely influenced by the time required to develop clinical trials; it is possible that trials started in the post-era were developed prior to guideline publication. Reassessment may be beneficial in the future as more modern-era trials report their results. Compliance with all guidelines may not be appropriate for every trial, particularly those with surgery or combination therapies that may have increased risk of adverse events yet target compliance rates haven’t been established. Last, it’s important to assess the impact of these changes in the accrued patient populations; however, only 33.7% (29 of 86) pre- and 3.6% (4 of 112) post-era trials reported results on clinicaltrials.gov at this time.

Although specific to PC, these data provide an understanding of modernized eligibility criteria use, demonstrating that great strides are still needed to improve diversity in PC clinical trials. Further investigation into their impact for other diseases is unknown and should be determined. With recent statements in support of increasing diversity in cancer clinical trials (15), we call for all involved to update protocols and scrutinize the need for restrictive criteria. We challenge all to critically assess eligibility criteria to promote diverse and equitable participation in cancer research and care.

## Supplementary Material

pkad009_Supplementary_Data

## Data Availability

The data underlying this article are available in the Zenodo data repository at https://dx.doi.org/10.5281/zenodo.7600042. The datasets were derived from sources in the public domain: ClinicalTrials.gov (https://clinicaltrials.gov).
