# Supplementary material for: Call to action: overcoming enrollment disparities in cancer clinical trials with modernized eligibility criteria
Source: JNCI Cancer Spectr. 2023 Feb 21;7(2):pkad009. doi: 10.1093/jncics/pkad009 (PMC9978314; doi:10.1093/jncics/pkad009)
Supplement: pkad009_Supplementary_Data [file pkad009_supplementary_data.pdf]

Supplementary Table 1. Compliance with modernized eligibility criteria by disease stage (unresectable, resectable, both), study phase, and study sponsor. Denominators vary based on availability of data; missing or unknown values were not included in determining the proportion of trials that were compliant. Allowance of brain metastases, if stable for > 4 weeks, was assessed only for trials that included patients with metastatic disease.

Eligibility Criteria	Disease Stage				Study Phase							
	Resectable	Unresectable	Both	p-value	Early 1	1	1|2	2	3	4	N/A	p-value
N (%)	62 (31.3)	115 (58.1)	21 (10.6)	--[a]	12 (6.1)	42 (21.2)	31 (15.7)	86 (43.4)	6 (3.0)	1 (0.5)	20 (10.1)	--[a]
Performance Status 2 Allowed	16/54 (29.6)	39/108 (36.1)	10/17 (58.8)	0.099	7/11 (63.6)	12/38 (31.6)	11/29 (37.9)	24/81 (29.6)	1/6 (16.7)	0/1 (0.0)	10/13 (76.9)	0.099
HIV+ Allowed	17/47 (36.2)	28/106 (26.4)	9/17 (52.9)	0.071	3/10 (30.0)	13/39 (33.3)	8/30 (26.7)	23/73 (31.5)	2/6 (33.3)	0/1 (0.0)	5/11 (45.5)	0.706
Renal Function Compliant (Use of CrCl only)	4/39 (10.3)	8/82 (9.8)	1/14 (7.1)	1.000	1/9 (11.1)	5/33 (15.2)	1/21 (4.8)	4/56 (7.1)	1/5 (20.0)	0/1 (0.0)	1/10 (10.0)	1.000
Reference Ranges for Labs	37/39 (94.9)	79/80 (98.8)	13/15 (86.7)	0.037	10/10 (100)	32/34 (94.1)	22/22 (100)	52/54 (96.3)	5/5 (100)	1/1 (100)	7/8 (87.5)	0.037
Classification System for Heart Failure	12/32 (37.5)	44/68 (64.7)	5/10 (50)	0.034	5/8 (62.5)	13/24 (54.2)	11/20 (55.0)	28/50 (56.0)	2/2 (100)	1/1 (100)	1/5 (20.0)	0.034
Cardiac Abnormalities Specified	33/62 (53.2)	81/115 (70.4)	12/21 (57.1)	0.059	9/12 (75)	26/42 (61.9)	24/31 (77.4)	57/86 (66.3)	3/6 (50)	1/1 (100)	6/20 (30.0)	0.059
QTc Compliant	53/56 (94.6)	104/108 (96.3)	19/19 (100)	0.730	11/11 (100)	39/39 (100)	30/30 (100)	74/81 (91.4)	6/6 (100)	1/1 (100)	15/15 (100)	0.730
Prior Malignancy >24 Months Ago Allowed	35/62 (56.5)	78/115 (67.8)	17/21 (81.0)	0.095	8/12 (66.7)	27/42 (64.3)	18/31 (58.1)	55/86 (64.0)	6/6 (100)	1/1 (100)	15/20 (75.0)	0.095
Concurrent Malignancy Allowed if Stable/Off Treatment	7/37 (18.9)	13/71 (18.3)	4/13 (30.8)	0.565	1/7 (14.3)	7/24 (29.2)	5/22 (22.7)	10/59 (16.9)	0/2 (0.0)	0/1 (0.0)	1/6 (16.7)	0.565
Prior Therapy Allowed & Exclusions Specified	22/23 (95.7)	95/97 (97.9)	10/11 (90.9)	0.276	6/6 (100)	34/36 (94.4)	24/25 (96.0)	52/52 (100)	¾ (75.0)	0/0 (0.0)	8/8 (100)	0.276
Time-Based Washout Period Used	10/22 (45.5)	60/99 (60.6)	4/11 (36.4)	0.158	4/6 (66.7)	17/36 (47.2)	20/26 (76.9)	27/52 (51.9)	¾ (75.0)	0/0 (0.0)	3/8 (37.5)	0.158
Recovery from Prior Adverse Events	3/22 (13.6)	47/99 (47.5)	4/11 (36.4)	0.011	1/6 (16.7)	8/36 (22.2)	13/26 (50)	28/52 (53.8)	¾ (75.0)	0/0 (0.0)	1/7 (12.5)	0.011
Brain Metastases Allowed if Stable >4 weeks	0/0 (0)	46/77 (59.7)	0/1 (0)	0.410	½ (50.0)	11/18 (61.1)	13/21 (61.9)	19/32 (59.4)	2/3 (66.7)	0/0 (0.0)	0/2 (0.0)	0.410

[a]Not applicable

Supplementary Table 1 (continued). Compliance with modernized eligibility criteria by disease stage (unresectable, resectable, both), study phase, and study sponsor. Denominators vary based on availability of data; missing or unknown values were not included in determining the proportion of trials that were compliant. Allowance of brain metastases, if stable for > 4 weeks, was assessed only for trials that included patients with metastatic disease.

Eligibility Criteria	Study Sponsor							
	Industry	Industry + NIH	Industry + Other	NIH	NIH + Other	Industry + NIH + Other	Other	p-value
N (%)	21 (10.6)	1 (0.5)	52 (26.3)	7 (3.5)	30 (15.2)	6 (3.0)	81 (40.9)	--[a]
Performance Status 2 Allowed	6/20 (30.0)	0/0 (0.0)	17/50 (34.0)	3/7 (42.9)	9/28 (32.1)	1/6 (16.7)	29/68 (42.6)	0.743
HIV+ Allowed	3/18 (16.7)	1/1 (100)	11/49 (22.4)	4/6 (66.7)	9/26 (34.6)	2/6 (33.3)	24/64 (37.5)	0.091
Renal Function Compliant (Use of CrCl only)	4/15 (26.7)	0/0 (0.0)	1/31 (3.2)	1/7 (14.3)	2/25 (8.0)	0/5 (0.0)	5/52 (9.6)	0.217
Reference Ranges for Labs	15/15 (100)	0/0 (0.0)	31/31 (100)	7/7 (100)	24/27 (88.9)	5/5 (100)	47/49 (95.9)	0.390
Classification System for Heart Failure	8/12 (66.7)	1/1 (100)	15/31 (48.4)	4/5 (80)	14/23 (60.9)	1/2 (50)	18/36 (50.0)	0.730
Cardiac Abnormalities Specified	14/21 (66.7)	1/1 (100)	32/52 (61.5)	6/7 (85.7)	24/30 (80.0)	2/6 (33.3)	47/81 (58.0)	0.152
QTc Compliant	16/18 (88.9)	1/1 (100)	45/47 (95.7)	6/7 (3.5)	27/28 (96.4)	6/6 (100)	75/76 (98.7)	0.181
Prior Malignancy >24 Months Ago Allowed	11/21 (52.4)	1/1 (100)	33/52 (63.5)	4/7 (57.1)	22/30 (73.3)	4/6 (66.7)	55/81 (67.9)	0.759
Concurrent Malignancy Allowed if Stable/Off Treatment	1/12 (8.3)	0/1 (0.0)	11/39 (28.2)	1/5 (20)	3/19 (15.8)	2/6 (33.3)	6/39 (15.4)	0.610
Prior Therapy Allowed & Exclusions Specified	16/17 (94.1)	0/0 (0.0)	42/42 (100)	6/7 (85.7)	22/23 (95.7)	5/5 (100)	36/37 (97.3)	0.196
Time-Based Washout Period Used	10/18 (55.6)	0/0 (0.0)	27/43 (62.8)	6/7 (85.7)	12/23 (52.2)	3/5 (60.0)	16/36 (44.4)	0.375
Recovery from Prior Adverse Events	11/18 (61.1)	0/0 (0.0)	16/43 (37.2)	5/7 (71.4)	12/23 (52.2)	1/5 (20.0)	9/36 (25.0)	0.034
Brain Metastases Allowed if Stable >4 weeks	8/15 (53.3)	0/0 (0.0)	18/28 (64.3)	3/4 (75.0)	8/11 (72.7)	0/1 (0.0)	9/19 (47.4)	0.552

[a]Not applicable
